# A Visual Tracker Offering More Solutions

**DOI:** 10.3390/s20185374

**Published:** 2020-09-19

**Authors:** Long Zhao, Mubarak Adam Ishag Mahmoud, Honge Ren, Meng Zhu

**Affiliations:** 1 College of Information and Computer Engineering, Northeast Forestry University, Harbin 150040, China; zhaolong@nefu.edu.cn (L.Z.); mubarakcom@nefu.edu.cn (M.A.I.M.); zhum913@163.com (M.Z.); 2 Big Data Institute, East University of Heilongjiang, Harbin 150066, China; 3 Forestry Intelligent Equipment Engineering Research Center, Harbin 150040, China

**Keywords:** visual tracking, neural architecture search, dual attention mechanisms, two-stage search

## Abstract

Most trackers focus solely on robustness and accuracy. Visual tracking, however, is a long-term problem with a high time limitation. A tracker that is robust, accurate, with long-term sustainability and real-time processing, is of high research value and practical significance. In this paper, we comprehensively consider these requirements in order to propose a new, state-of-the-art tracker with an excellent performance. EfficientNet-B0 is adopted for the first time via neural architecture search technology as the backbone network for the tracking task. This improves the network feature extraction ability and significantly reduces the number of parameters required for the tracker backbone network. In addition, maximal Distance Intersection-over-Union is set as the target estimation method, enhancing network stability and increasing the offline training convergence rate. Channel and spatial dual attention mechanisms are employed in the target classification module to improve the discrimination of the trackers. Furthermore, the conjugate gradient optimization strategy increases the speed of the online learning target classification module. A two-stage search method combined with a screening module is proposed to enable the tracker to cope with sudden target movement and reappearance following a brief disappearance. Our proposed method has an obvious speed advantage compared with pure global searching and achieves an optimal performance on OTB2015, VOT2016, VOT2018-LT, UAV-123 and LaSOT while running at over 50 FPS.

## 1. Introduction

Visual target tracking has important applications in areas such as autonomous driving, intelligent security, human computer interaction and robotics [1,2,3]. A complete tracking system consists of three components: a search strategy, the feature extraction and an observation model. The continuous development of deep learning technology, particularly the gradual maturity of AutoML methods, provides new opportunities for visual target tracking research. However, tracking is a serialization problem and faces multiple challenges, including variations in lighting and scale, occlusion, background interference, rotation and motion blurring. Currently, several fully convolutional Siamese network trackers (e.g., SiamRPN++ [4], SiamMask [5] and SiamDW [6]) based on offline end-to-end training have achieved robust performance on the VOT2018 [7], UAV123 [8] and GOT10K [9] datasets. Yet these methods only employ offline learning, with target template models generally focusing on apparent modelling, resulting in poor performance for occlusion and highly similar target interferences. Moreover, online-only approaches (e.g., C-COT [10] and ECO [11]) lack model generalization and thus perform poorly in target regression. The networks of such approaches require constant fine-tuning, preventing real-time tracking requirements to be met. From the perspective of methods, in addition to the Siamese network-based methods that have dominated in recent years (e.g., [12]), a research branch began to focus on small sample learning target tracking methods represented by Meta Learning (e.g., [13,14]), with another research branch always insisting on the use of correlation filter approaches (e.g., [15,16,17,18]). Ocean [12] represents the trackers based on a Siamese network evolved from Anchor-Based to Anchor-Free. The core of the Meta Learning is to let the machine learn to learn. The target tracking method based on Meta Learning uses meta-learning to optimize adaptively the target tracking model, so that the model can adapt quickly to different video sequences or scenes. Although Meta Learning is of great research value in target tracking tasks, current methods based on Meta Learning have poor performance when the background is complex. DiMP [17] introduced Meta Learning after ATOM [16] to update the template. PrDiMP [18] proposed a probabilistic regression formulation and applied it to tracking. Although DiMP and PrDiMP have improved on ATOM, it should be ATOM that really brings correlation filter approaches back to the top.

In order to achieve the speed requirements while also improving tracker accuracy and robustness, Danelljan et al. proposed ATOM with dedicated target estimation and classification components. In particular, due to the high complexity of target estimation, the target estimation component of ATOM is trained completely offline on large-scale datasets, while classification components are trained online to ensure excellent discrimination in the presence of interference factors. ATOM also uses gradient ascent to maximize the IoU score for the final bounding box during the tracking process. Compared with related methods, ATOM has been able to achieve improvements in precision of up to 15% on the TrackingNet [19] dataset, as well as great accuracy improvements on the VOT2018 [7], Need for Speed (NFS) [20] and UAV123 [8] datasets for speeds exceeding 30 FPS.

Despite its great success in visual tracking tasks, the feature extraction capability of the ATOM backbone network (ResNet18) is not strong enough. During the training process, the maximum Intersection-over-Union (IoU) is used to estimate the position of the target with a slow and unstable network convergence. ATOM performs poorly when faced with the sudden rapid movement of the target and the recurrence of the target following its disappearance. Such challenges are very common in long-term tracking tasks. Thus, we propose a tracking method that is both accurate and robust for long-term visual target tracking. We employ neural architecture search technology as the backbone of the tracker and maximize the Distance Intersection-over-Union (DIoU) [21] to estimate the target accurately. The tracker discriminator is increased with the inclusion of CBAM [22], while we improve the tracker speed by using the two-stage search (TSS) method combined with a screening module (SM).

The key contributions of this paper can be summarized as follows:

i. We present the first attempt to use EfficientNet-B0 [23] via neural architecture search technology as the backbone network. We achieve the top-ranking performance while significantly reducing the backbone network parameters.
ii. We propose a novel tracking framework with DIoU estimation as the core. Our target estimation component is trained to predict the DIoU between the target object and an estimated bounding box. This allows faster converge and greater stability during training. The DIoU is employed as a guide for Non-Maximum Suppression (NMS) operations when suppressing redundant detection frames.
iii. We add CBAM to the target classification module and generate attention maps on the channel and spatial dimensions to perform constraint enhancement processing on the original features. This improves significantly the discrimination of the tracker.
iv. We use a two-stage search method to solve the challenges associated with long-term tracking tasks. During the second search step, the screening module is able to identify quickly the areas where targets are most likely to exist. The screening module improves not only the speed of the tracker, but also the tracker robustness via the interference filtering.

## 2. Related Work

In general, visual tracking methods can be classified into two types in terms of their observation models: generative models and discriminative models. A key limitation of generative model-based methods is the omission of background information. In contrast, discriminative models consider both target and background information, and complete the tracking task by determining a discriminant function to separate the target from the background. Moreover, the majority of such models employ classification and regression to complete the target judgment. On the whole, these two approaches are slowly converging with the advancement of research. The correlation filter [24,25,26,27,28,29] approach is a commonly used discriminative model, which improves the speed via the fast Fourier transform for time-to-frequency domain conversions. The application of deep learning methods in visual basic tasks (e.g., image classification and target recognition), with powerful representation capabilities for deep features, has proved to be extremely successful in recent years. Gradually, deep learning has become a mainstream feature in tracking methods. Consequently, the latest correlation filter methods [30,31] make use of depth features due to their powerful representation capabilities.

The number of samples obtained during tracking is limited though substantial training samples are compulsory for deeper network training. Hence, CNN-based tracking methods generally pre-train networks offline. Then, tracking results fine-tune the online network to adapt to changes in the target and background of the current tracking video based on the labelling of the first frame and subsequent frames during tracking. Nam et al. [32] proposed a training method in a multi-target domain, whereby the training network consists of a shared network layer and multiple specific layers. Their follow-up work [33] determined the optimal tracking model by learning multiple depth models and constructing a tree structure. Song et al. [34] introduced adversarial learning to simulate occlusion samples by generating an occlusion mask. These samples were applied during classifier training to improve the classifier’s robustness to occlusion. In the literature, [35] specifically designed a CNN to identify whether the target is occluded; however, [35] did not solve the fundamental occlusion problem in tracking, and the speed is very slow.

Visual tracking tasks require not only accurate prediction of the target’s position, but also to at least equal the tracking speed to the frame rate of the video, if not greater. SiamFC [36] achieves an excellent performance while achieving speeds of 80 FPS. Compared with SiamFC, SiameseRPN [37] has increased the scale estimation and performed better when the target aspect ratio changes. Hence, the speed is also faster, reaching a top of 160 FPS. These Siamese methods regard the visual target tracking as learning a general similarity map through the cross-correlation between the target template and the feature representation learned from the search area. In order to ensure tracking efficiency, the similarity metric function for offline learning is often fixed at runtime [36,38]. CFNet [39] and DSiam [40] update the tracking model by running an average template and a fast conversion module, respectively. SiameseRPN introduced a region proposal network following the Siamese network, combining classification and regression for the tracking process. DaSiamRPN [41] extended this by introducing an interference perception module, improving the model’s recognition capabilities. Siamese network-based methods have recently made breakthrough progress; in particular, the Siamese trackers [4,5,6], breaking the network depth limit, has achieved a state-of-the-art performance on several visual tracking datasets. However, directly using deeper networks (e.g., ResNet [42] and Inception [43]) as the tracker backbone network does not improve the tracker performance. In fact, the deeper the network is, the poorer the performance shows [6]. AlexNet [44], the backbone network of SiamFC and SiameseRPN, trimmed all layer padding, while ResNet and Inception require padding to ensure the required depth of the network. As for the deepening of the network, the receptive field increases sharply. The searching region exceeds the image boundary during the searching of the target for it could probably appear anywhere. The application of padding in the network consequently removes the absolute translation invariance. Thus, the one-to-one correspondence between the positions of the maximum response value in the feature maps and the large response value in the original map will be destroyed. This consequently results in the failure of the tracking task. SiamRPN++ [4] and SiamDW [6] overcome this problem through a spatially aware sampling strategy and by including a cropping-inside residual module following a common residual unit, respectively. The literature [45] proposed a visual target tracking framework based on a cascaded RPN; however, the speed is slower although the robustness and tracking accuracy of this framework are greatly improved.

Current tracking methods are not able to fully solve the occlusion problem. Thus, GCT [46] proposed a graph convolution tracking framework that can simultaneously model the spatiotemporal appearance of the target and perform context-aware adaptive learning under a unified framework, followed by the robust positioning of the target. GCT solves the occlusion problem by reducing the weight of the occluded component in the input information of the target appearance model.

Sudden target movements and the reappearance of a target following its disappearance increase the difficulty of the tracking tasks. In order to recapture the target in such cases, Zhang et al. [47] combined local and global search strategies. In addition, inspired by two-stage object detectors, Zhang et al. [48] considered tracking as a fully global instance search task whereby the tracking process is constant for all frames following the determination of the target frame. However, both prove to be extremely slow (neither exceed 6 FPS) and are thus not even close to meeting real-time requirements.

Deep DCNN features impose limitations on target tracking tasks requiring high positioning due to its low spatial resolution and poor positioning capabilities, despite their stronger semantic resolution capabilities. In order to solve this problem, [49] improved the Hedge algorithm, an online decision theory for combining the DCNN multi-layer features. In this way, the shallow DCNN features with a higher resolution and conducive to spatial positioning can complement each other with the deep DCNN features with strong semantic discrimination.

## 3. Materials and Methods

**Figure 1:**
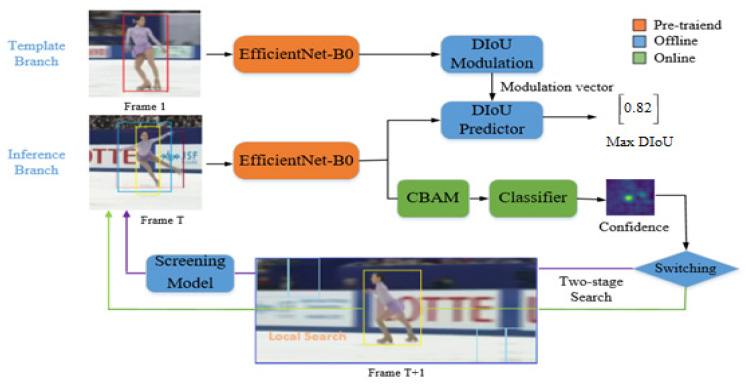
Overview of the proposed network architecture for visual tracking.

Figure 1 presents an overview of the framework proposed in this study. Four key components are employed here: (i) The selection of the tracker’s backbone network; (ii) an accurate target estimation; (iii) a target and background discrimination approach; and (iv) the recapturing of the target.

### 3.1. New Backbone Network

With the constant development of AutoML technology, research into Neural Architecture Search (NAS) has achieved promising results, such as EfficientNet, the baseline network designed by Google [23]. EfficientNet has demonstrated prominent accuracy and efficiency compared to previous convolutional networks. In particular, EfficientNet-B7 is able to achieve the highest top-1 and top-5 accuracies of 84.4% and 97.1%, respectively, on ImageNet, and is also 8.4 times smaller and 6.1 times faster than the previous optimal convolutional network. When EfficientNet-B0’s top-1 accuracy and top-5 accuracy are all higher than ResNet-50, the Floating-point Operations Per Second (FLOPS) is only 1/11, and the parameters are only about 1/5. As well as great success in image classification, NAS technology has also excelled in object detection. For example, EfficientDet [50] employs EfficientNet as the backbone network and combines new feature fusion methods (BiFPN) to achieve a state-of-the-art performance. EfficientDet-D7 achieved a 51.0 mAP on the COCO 2017 validation dataset with 326 B FLOPS and 52 M parameters.

Visual tracking is regarded as a serialization detection task and thus EfficientNet-B0 (Table 1) can be applied naturally as the tracker backbone network for feature extraction. Existing depth trackers generally use deep features pre-trained by convolutional neural networks for visual tracking. EfficientNet will still respond to objects detected in the pre-training process, similar to classification networks such as ResNet. The aim of the tracking task is to distinguish the target from the background, and must be able to overcome the interference of different and similar object types on the target. Traditional classification networks focus on the differences between classes but demonstrate a weak ability in distinguishing differences within classes, whereas EfficientNet proves strong discrimination capabilities in both situations.

**Figure 2:**
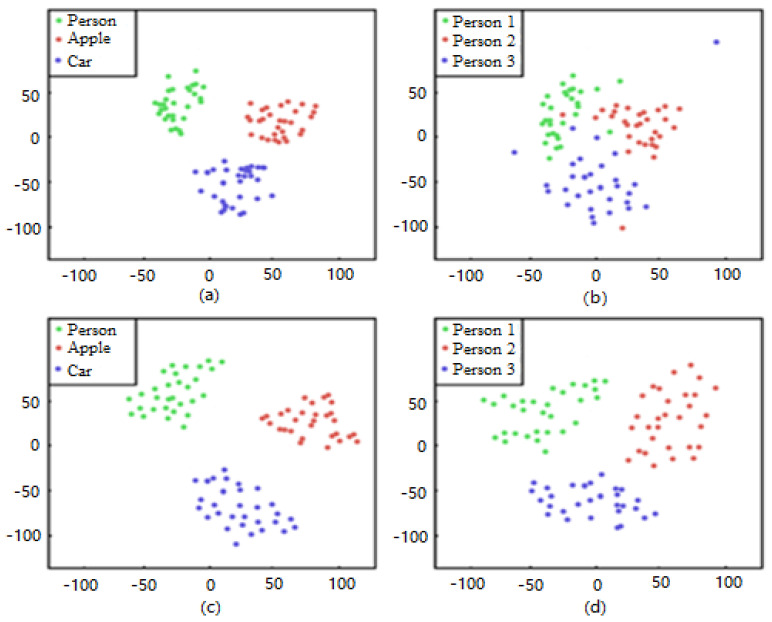
Pre-trained features using the t-SNE method. In (**a**) and (**c**) the differently colored points belong to different object classes; In (**b**) and (**d**) all points belong to the person class but in different videos.

A total of 30 videos were randomly selected for the experiments in the current study. Figure 2 presents the t-SNE method [51], which was applied for data reduction to two-dimensional space. Each point denotes a target in one frame. In Figure 2a,c, the differently colored points belong to different object classes, while in Figure 2b,d, all points belong to the person class but in different videos. In addition, Figure 2a,b are pre-trained features by ResNet50 and Figure 2c,d are pre-trained by EfficientNet-B0.

Figure 2a,c demonstrates the slightly stronger ability of EfficientNet-B0 compared to ResNet50 in discriminating different types of targets. By comparing Figure 2b with Figure 2d, we can see that EfficientNet-B0 has more obvious advantages in discriminating different instances of the same object class.

### 3.2. Target Estimation by DIoU Maximization

We first train the network offline on a large number of datasets. This is performed in order to accurately estimate the framed target of the initial frame in subsequent video sequences. Template branch and inference branch share convolutional features by the Siamese network. Considering its ability to extract more accurate RoI features on the feature map, Precise RoI Pooling [52] is employed rather than the commonly used RoIPooling. Precise RoI Pooling is an integration-based (bilinear interpolation) average pooling method for RoI Pooling that avoids quantization and has a continuous gradient on bounding box coordinates.

We define the deep feature representation of an image, x ∈ ℝ^W×H×D^, and a bounding box estimate B ∈ ℝ^4^ of an image object. Here B is parametrized as B = (c_x_/w, c_y_/h, log w, log h), while (c_x_, c_y_) are the image coordinates of the bounding box center. We use a Precise ROI Pooling layer to pool the region in x given by B. This generates feature map x_B_ of a predetermined size. Our goal is to maximize the DIoU between bounding box B and the ground truth. B is refined as the DIoU increases. For computational efficiency, we formulate it as a DIoU loss.

The reason why we do not maximize IoU like ATOM is because using IoU makes the network offline learning convergence slow and the convergence process is unstable. The IoU is determined as in Equation (1), and is subsequently used to derive the IoU loss in Equation (2):

(1) $$IoU=\frac{\left|B\cap B^{gt}\right|}{\left|B\cup B^{gt}\right|}$$

(2) $$L_{IoU}=1-\frac{\left|B\cap B^{gt}\right|}{\left|B\cup B^{gt}\right|}$$

Experiments demonstrated that the application of IoU for the bounding box regression resulted in a closer distance of the anchor to the edge and, consequently, the larger error. In addition, IoU loss only works when the bounding boxes have an overlap and would not provide any moving gradient for non-overlapping cases. Thus, we introduced the DIoU, initially proposed by [21]. The DIoU loss is a penalty term introduced on the basis of the IoU loss, as described in Equation (3):

(3) $$L_{DIoU}=1-\mathrm{IoU}+\frac{\rho^{2}b,b^{\mathrm{gt}}}{c^{2}}$$

where b and b^gt^ are the center points of B and B^gt^, respectively, and ρ is the Euclidean distance and c is the smallest length of the diagonal covering B and B^gt^. The penalty minimizes the center point distance d.

**Figure 3:**
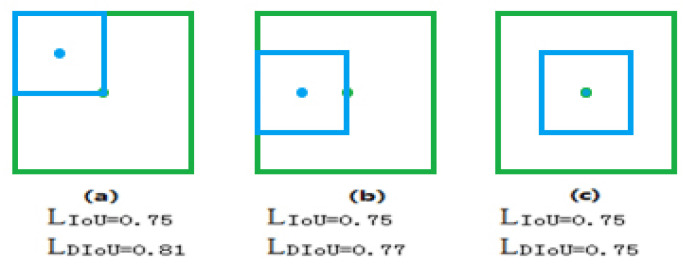
Intersection-over-Union (IoU) and Distance Intersection-over-Union (DIoU). Green and blue denote the target box and predicted box, respectively. (**a**) The predicted box is in the upper left corner of the target box; (**b**) The predicted box is on the left side of the target box; (**c**) The center of the predicted box overlaps the center of the target box.

From Equations (1) and (2), we can see that if the value of IOU does not change, the value of L_IoU_ does not change. Yet due to the overlapping positions of the predicted and target boxes, the DIoU loss is also different (Figure 3). The DIoU loss can still provide a moving direction for the bounding box for non-overlapping predicted and target boxes. In addition, as the DIoU loss can directly minimize the distance between predicted and target boxes, it converges much faster than the IoU loss. By comparing Figure 3a–c, we can see that despite having equal IoU values, Figure 3c is more favorable for determining the search position of the next frame. We use the DIoU to replace the standard IoU evaluation strategy in the NMS process, increasing the accuracy and effectiveness of the NMS results.

The template branch inputs features *x*_0_ and target bounding box annotation B_0_ in the first image. Similar to ATOM, it returns a modulation vector v(*x*_0_; *B*_0_) consisting of positive coefficients of size 1 × 1 × D_z_. In the inference branch, we estimate the bounding box of the target in the current frame (Frame T). Two convolutional layers are used to feed the backbone feature *x*_t_, while the Precise ROI Pooling is combined with the bounding box estimate *B_t_* to extract the depth representation. The size of the determined representation z(*x*_t_; *B*_t_) is K × K × D_z_, where K is the spatial output size of the Precise ROI Pooling layer. The search image used to compute the feature representation is then modulated by coefficient vector *v* via channel-wise multiplication. Module g is subsequently used for DIoU prediction by effectively integrating the appearance information of *v* and z. The predicted DIoU of bounding box *B_t_* of the T-th frame image is described in Equation (4).

(4) $$DIoU\left(B_{t}\right)=g\left(v\left(x_{0},B_{0}\right)\cdot \;z\left(x_{t},B_{t}\right)\right)$$

### 3.3. Improved Target Discrimination via CBAM

We do not deny that the Siamese trackers represented by SiamMask [5] can achieve a state-of-the-art performance on numerous datasets. However, these trackers are easily distracted by similar objects during the tracking process. This is attributed to the lack of online weight updating for background noise suppression. Although the target estimation module provides an accurate bounding box output, it lacks the ability to distinguish target objects from the background like the Siamese tracker. Therefore, we use the second network header as the target classification module for online training, with the sole purpose of performing this discrimination. The output of the classification module is a confidence score of the target.

**Figure 4:**
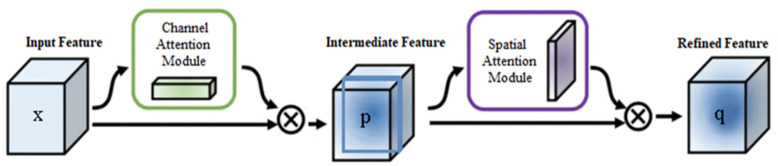
The convolution block attention module.

Inspired by ATOM [16], we use a two-layer fully convolutional neural network to implement the target classification module. However, in order to enhance the discriminative ability of our target classification module, we include CBAM [22] into the target classification module. As demonstrated in Figure 4, we generate attention maps on both the channel and spatial dimensions to constrain the input features, where x is the backbone feature map. The two key operations performed by CBAM are shown in Equation (5):

(5) $$\begin{array}{c} p=M_{c}(x)⊗x\\ q=M_{S}(p)⊗p \end{array}$$

where ⊗ denotes element-wise multiplication. First, the channel attention map is multiplied with input x to obtain p. The spatial attention map of p is then calculated and multiplied with p to obtain final output q, the input of the target classification module. The fully convolutional neural network is defined as Equation (6):

(6) $$f\left(q;w\right)=\emptyset_{2}\left(w_{2}*\emptyset_{1}\left(w_{1}*q\right)\right)$$

where ∗ denotes the convolution operation, $\emptyset_{1}$ and $\emptyset_{2}$ are activation functions and $w_{1}$ and $w_{2}$ are the network parameters. Following ATOM, we formulate a similar learning objective based on the L^2^ classification error, as described in Equation (7):

(7) $$L\left(w\right)=\overset{n}{\underset{i=1}{\sum }}r_{i}\|f\left(q_{i};w\right)-y_{i}{\|}^{2}+\underset{\dot{J}}{\sum }\lambda_{j}\|w_{j}{\|}^{2}$$

where $q_{i}$ indicates the refined feature following CBAM processing and f(q;w) denotes the classification score for each location belonging to the target region and $\;r_{i}$ represents the weight of each training sample. Based on the given target bounding box, we set $y_{i}$ to a Gaussian distribution. The regularization amount of $\;w_{j}$ is set to $\lambda_{j}$. As online learning requires rapid network convergence, it can thus meet the real-time requirements of trackers. The conjugate gradient (CG) algorithm proposed by ATOM [16] was employed as the optimization strategy.

### 3.4. Enhancement of Tracker Speed by TSS and SM

**Figure 5:**
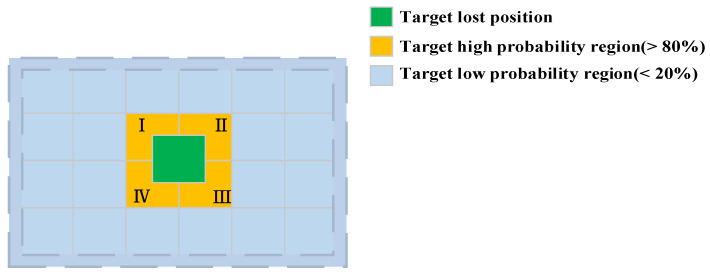
Probability of the target appearing in difference regions of the image.

It is hard for trackers such as ATOM and SiamRPN++ to recapture the target once it fails to be positioned and moves out of the search range. These trackers also fail to identify targets rapidly following their brief disappearance from the field of view and subsequent reappearance. In order to overcome such challenges, [47] fused local and global search strategies. Ref. [48] inspired by their two-stage target detector Faster R-CNN. As a global instance search task, the tracking is fulfilled if the target frame of the first frame is provided, after which the tracking process for each subsequent frame is the same [48]. Although the global search strategy used by [47,48] can solve the aforementioned problems, it is too slow to meet the real-time requirements of tracking. Experimental results demonstrated the spots of reappearance of the target after sudden movements or brief disappearances can be traced. Figure 5 presents the probability of the target appearing in different regions of the image for the VOT and OTB datasets. For more than 80% of cases, it is not necessary to search the light blue area. Meanwhile, the probability of a global search on these data sets does not exceed 20%. Thus, the global search should be fully planned in order to avoid its inefficient utility.

In order to overcome this limitation, we proposed the TSS method. In brief, at the first stage of the search, we use the sliding window method in areas where the target is likely to be sent to the inference branch one by one (I–IV) for inference. If the target is not detected at the first stage, the second stage search is initialized. At this stage, the SM is used for areas with low probabilities of target appearance, while the first K areas with the highest probability are screened and sent to the inference branch for further inference. This process is explained in more detail in the following.

**Figure 6:**
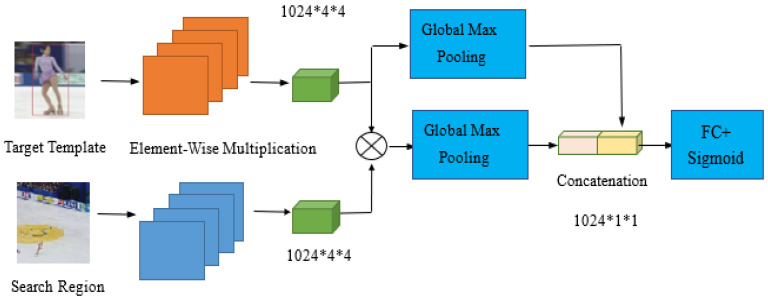
Network architecture of the proposed screening module.

If the output confidence score of the target classification module is less than the threshold Ω (Ω = 0.5), the tracker fails at the T-th frame. The first search stage is then initiated, and the search area expands to four times the enlarged inference branch input image during the T + 1 frame tracking (orange area in Figure 5). A sliding window is used to take I-IV as the input of the inference branch and the target position is taken as the maximum confidence score position in the orange area. If the maximum confidence score is greater than Ω, we no longer perform the second stage search, and the entire search process ends. If the maximum confidence score is still less than Ω, we discard the orange area as the location of the target and start the second stage search in the next frame. The sliding window technique was not employed during the second stage of the search process as it is extremely time-consuming, particularly for deep-learning-based models [37,53]. The larger the image size, the more time-consuming the sliding window. We define the problem as Equation (8):

(8) P=u(Z,X)

where Z is the target template, X is the search area, u is a similarity measure function, and P is the probability that the target appears in area X. We use a simple deep convolutional neural network to implement this function, and denote it as a screening module. Screening modules aim to learn a function P = u(Z, X). Figure 6 depicts the network architecture. In order to improve the accuracy of the prediction, we apply cross entropy loss during the training of our network.

The SM is able to determine areas quickly in the light blue regions where targets are potentially located. In particular, only the first K (K = 2) regions are most likely to have targets sent to the inference branch, while the remaining regions are discarded. With its filtering of interference, the SM can enhance the tracker’s robustness, not just increasing speed.

## 4. Experiments and Discussion

We implement the proposed tracker in Python3.6 with PyTorch on a PC with the following specifications: 16 GB memory, i7 8700K 3.7 GHz CPU and a GTX-1080Ti GPU. Our experimental environment is equal to the development environment.

### 4.1. Implementation Details

Network Architecture: EfficientNet-B0 is used as the backbone network of the tracker. The displacement of the target between two adjacent frames is generally not very large in tracking tasks. Thus, the stride of our backbone network is too large to affect target positioning and we reduce the effective strides at the final stage (stage three) from 16 pixels and 32 pixels to 8 pixels by modifying the stage 6, stage 7 and stage 8 blocks to have a unit spatial stride, and also increase their receptive field by dilated convolutions. Visual tracking requires rich features; even with deep features in convolutional networks, separate layers are not enough. Low-level features contain more location information, while higher-level features have richer semantic information. Compounding and aggregating these features can improve the recognition and localization of targets. We use the feature outputs of stage 5, stage 6 and stage 8 as the subsequent input, which can significantly improve the performance of the tracker.

Offline Training: We train the backbone network offline using the COCO [54], TrackingNet [19], Youtube-BB [55] and GOT10K [9] training sets. We sample image pairs from the video at a maximum interval of 120 frames. COCO and Youtube-BB images are used to synthesize image pairs, increasing the diversity in categories of our training data. We perform rotation and blurring data augmentation processes on the first frame. The template and inference branch input images have dimensions of 224 × 224. For training data pairs, we use Gaussian noise to generate 16 candidate bounding boxes with a minimum DIoU of 0.15 to ground truth coordinates. Following ATOM, the weights in our head network are initialized by using [56]. During training, the weights of the backbone network are frozen, and the mean-squared error loss is used for optimization, and per batch, which contain 64 image pairs training 40 epochs. The initial learning rate is set to 0.01, and is subsequently reduced by 0.02 every 20 epochs.

Online Learning: The channel and spatial dual attention mechanisms are applied to improve the discrimination of the target classification module. In addition, the conjugate gradient method is used to increase the speed of online learning. We reformulate Equation (7) to express the squared norm of the residual vector $\;L\left(w\right)=\|r\left(w\right)\|{\;}^{2}$, where $r_{i}\left(w\right)=\sqrt{r_{i\;}}\left(f\left(q_{i}\right)-y_{i}\right)$ and $\;r_{n+j}\left(w\right)=\sqrt{\lambda_{j}}w_{j}$, Thus, we have a positive definite quadratic problem.

### 4.2. Comparison to State-of-the-Art Trackers

**Figure 7:**
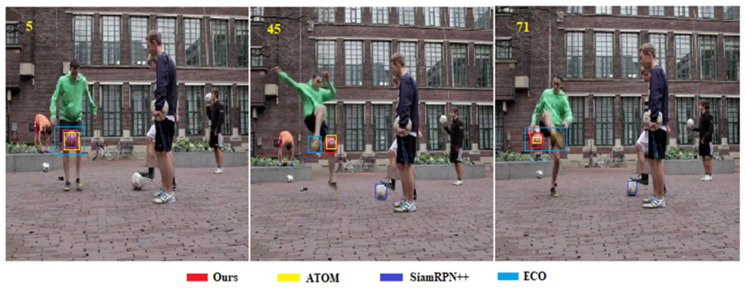
Short video comparison of the proposed framework with state-of-the-art trackers.

Short video comparison: A video from VOT2016 [57] was selected to qualitatively compare our approach with state-of-the-art trackers. Our tracker outperforms the other three trackers both in accuracy and robustness (Figure 7; the yellow number represents the sequence of the current frame). The performance of ATOM is closest to that of our proposed method. Although SiamRPN++ is also highly accurate, the interference of similar objects is frequently observed. ECO exhibits the worst performance.

**Figure 8:**
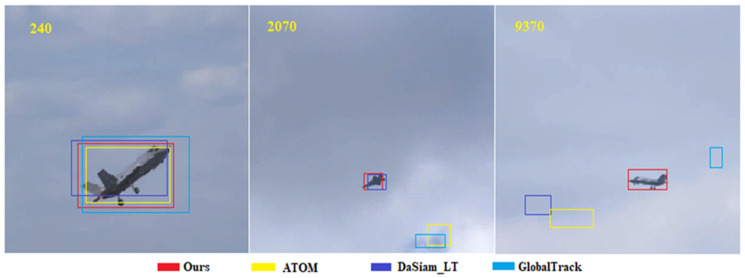
Long video comparison of the proposed framework with state-of-the-art trackers.

Long video comparison: A video with a length of 11,397 frames from the LaSOT [58] dataset was used to compare trackers for long-term tasks. Figure 8 demonstrates that frame 9730 can only be captured using our tracker (Figure 8). Despite its high accuracy, ATOM does not have a global search capability, thus the target is lost at frame 2070. In addition, GlobalTrack [48] treats tracking as a global instance search problem; that is, global detection is performed for each subsequent frame following the provision of a target frame for the first frame. At 6 FPS, its maximum speed is far from real-time speed. Although DaSiam_LT has real-time and global search capabilities, it does not have the ability to learn online. This seriously reduces its discrimination results.

**Figure 9:**
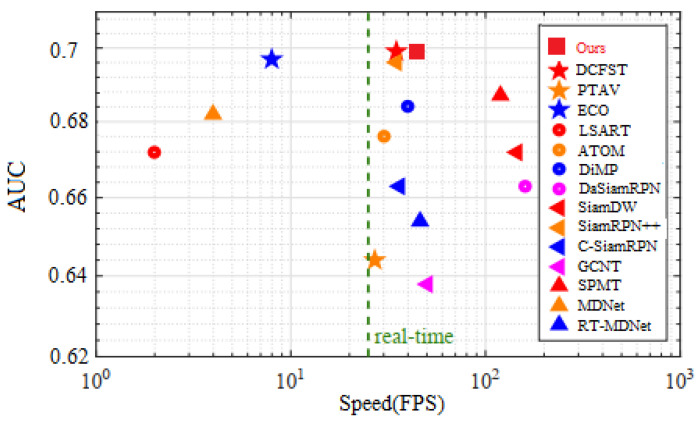
Comparison between the proposed framework and state-of-the-art tracking algorithms using the OTB2015 benchmark.

OTB2015 Dataset: OTB2015 [59] contains 100 video sequences, ranging from tens of frames to more than 3000 frames in length. The entire database has a total of 58,897 frames. OTB2015 uses success plots, the mean overlap precision and Area Under Curve (AUC) to quantitatively evaluate the performance of all trackers. Figure 9 compares our tracker with 14 state-of-the-art trackers in terms of AUC and speed. The AUC of our tracker almost reaches that of DCFST [60] at a greater speed. Thus, our method is able to achieve an optimal balance between accuracy and speed.

**Figure 10:**
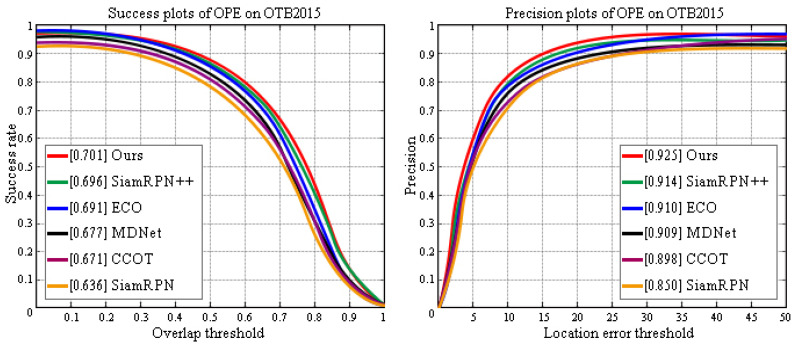
Success and precision plots show a comparison of our tracker with state-of-the-art trackers on the OTB2015 dataset.

In order to verify that the two indicators of precision and success plots also have advantages on OTB2015, we compared our method with five other classic target tracking frameworks. It can be seen from Figure 10 that our proposed framework achieves the optimal performance.

VOT2016 Dataset: VOT2016 contains 60 video sequences. Compared with the previous version of VOT, VOT2016 used the method of automatically labeling samples to relabel the samples without expanding the sample set. We adopt the Expected Average Overlap (EAO), Accuracy (A) and Robustness (R) measures to compare the trackers. Compared with the other four frameworks, such as C-RPN [45], our proposed framework tops in all performance indicators. Compared with the SiamDW and C-RPN approaches, our method achieves a performance relative gain of 15.9% and 18.2%, respectively. The specific results are shown in Table 2.

VOT2018 Dataset: The trackers are further compared using the VOT2018 dataset [7], adding speed (fps) (Table 3). The VOT2018 benchmark contains a greater number of challenging factors compared to the OTB series dataset, and can thus be considered as a more comprehensive test platform in terms of accuracy and robustness. Our proposed method achieved the runner-up performance on EAO and Robustness indicators, second only to Ocean [12]. But our accuracy indicator is higher than Ocean’s one, and the speed is more than twice that of Ocean (online). Compared with the SiamRPN++ and ATOM approaches, our method achieves a performance relative gain of 2.7% and 6%, respectively. Moreover, our tracker demonstrated the second highest accuracy value, with SiamMask achieving the maximum result. The output expression of the mask proposed by SiamMask contributes to the high accuracy of the tracker index.

VOT2019 Dataset: Compared with four latest methods proposed by Ocean, MAML [61], SiamBAN [62] and SiamDW on the basis of the VOT2019 dataset [63], our method achieved sub-optimal performance on all performance indicators. The EAO index of our method is second only to Ocean (online), but the speed is two times that of Ocean (online). The accuracy index of MAML performs best, which also shows that Meta Learning is feasible in target tracking tasks. The specific results are shown in Table 4.

GOT10K Dataset. In the GOT0K dataset, more than 1.5 million bounding boxes were manually labeled. These video sequences are divided into 563 target categories and 87 motion modes. The targets are all moving objects in the real world. The dataset is divided into three subsets: training, verification and testing. There are many benefits of clear separation between the training set and the test set. This can not only increase the fairness of the test, but also verify the generalization ability of the model. We have done comparative experiments with the three latest trackers in the test subset. It can be seen from Table 5 that we have achieved the best performance on the AO index and the sub-optimal performance on the SR_0.5_ index.

**Figure 11:**
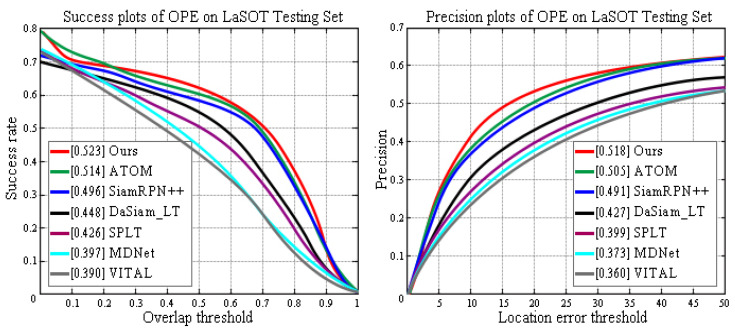
Comparison between the proposed framework and state-of-the-art tracking algorithms using the LaSOT benchmark.

LaSOT Dataset: LaSOT [58] contains 1400 videos with an average of 2512 frames per sequence. The dataset has 70 categories, each with 20 sequences. LaSOT is by far the largest object tracking dataset with high quality manual dense annotations. Figure 11 compares our framework with the most advanced trackers using the LaSOT dataset. Compared with the previous optimal tracker (ATOM), our approach achieves relative gains of 2.5% and 1.7% in the precision and success scores, respectively. These results prove the advantages of dynamic area searches in long-term tracking.

**Figure 12:**
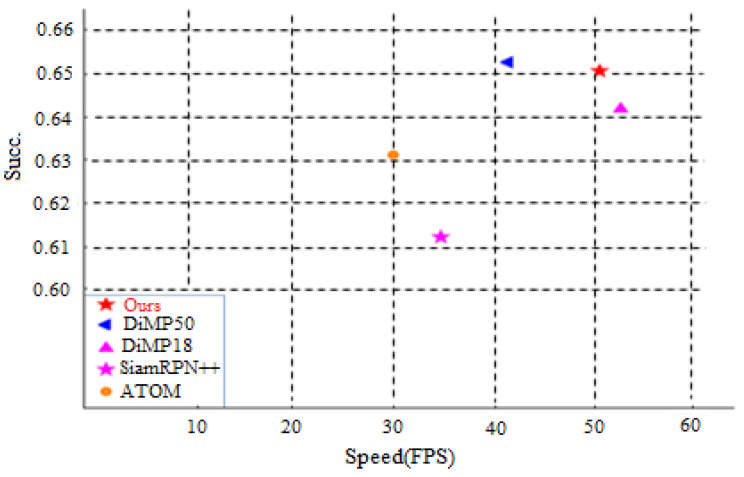
Comparison between the proposed framework and state-of-the-art tracking algorithms using the UAV-123 benchmark.

UAV-123 Dataset: The UAV-123 dataset compromises scenes shot with drones, with clean backgrounds and highly variable viewing angles. With a total of 123 videos, the size of the dataset is approximately 13.5 G. Figure 12 compares our tracker with four state-of-the-art trackers in terms of success rate and speed. The success rate of our tracker is slightly lower than that of DiMP50 [17], yet its speed is higher. Moreover, although DiMP18 is faster than our tracker, its success rate is lower. Our method thus achieves an efficient balance between accuracy and speed.

**Figure 13:**
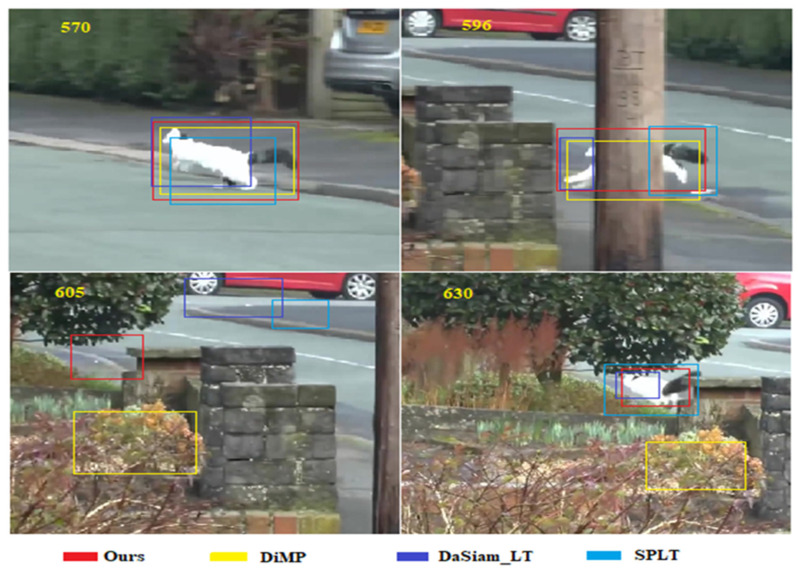
Comparison between the proposed framework and state-of-the-art tracking algorithms using a video from VOT2018-LT.

VOT2018-LT Dataset: VOT2018-LT is generally used to evaluate the performance of trackers on long-term targets. Unlike the corresponding short-term tracking dataset, in VOT2018-LT, it is common for the target to temporarily disappear in the view range for a period of time. The target then reappears in a random location, which requires the tracker to have a global search capability to recapture the target quickly. Figure 13 compares our tracker with the recently proposed advanced trackers DiMP and SPLT [64], as well as DaSiamRPN [41], using a video sequence from the VOT2018-LT dataset. DiMP, with the same online learning ability as our tracker, exhibits a significantly higher accuracy than that of SPLT and DaSiamRPN using offline learning. However, this may not be important in long-term tracking. In particular, after the target disappeared in frame 605, DiMP (without a global search capability) was the only tracker not able to recapture the target following the reappearance of frame 630. This proves the importance of the global search capability in long-term tracking.

### 4.3. Ablation Study

A comprehensive ablation study was performed by using the VOT2018 dataset in order to demonstrate the impact of each component of our proposed method.

Backbone Architecture: The backbone network is used for feature extraction and forms the basis of a tracker. The number of backbone network parameters and the type of layers directly affect the tracker’s memory, speed and performance. At present, the most advanced trackers generally use ResNet-50 as the backbone network. In this paper, we demonstrate for the first time the application of EfficientNet-B0 using neural architecture search technology as the backbone network. From Table 3, we can find that when other modules are the same, using EfficientNet-B0 as the backbone network, the EAO of the tracker can be increased by up to 3%. Furthermore, EfficientNet-B0 requires just 1/5 of the parameters used by ResNet-50 [23].

DIoU/IoU: Table 6 reports the effect of maximizing the IoU and DIoU to estimate the target position in the estimation module. The DIoU loss introduces a penalty term based on the IoU loss that can minimize the distance between the bounding box and the center point of the ground truth. The relatively accurate prediction of the target center point is helpful for tracking this sequence of words detection tasks. The EAO of the tracker with the DIoU is greater than that with the IoU. In addition, using the DIoU reduces the offline training time by 50%.

CBAM: CBAM [22] generates attention maps on the channel and spatial dimensions to perform constraint enhancement processing on input features (Table 6). Employing CBAM on the target classification module of the tracker results in a 1.5% improvement on EAO. This further proves that the attention mechanism is not only suitable for basic computer vision tasks, but can also be applied to tracking tasks.

TSS + SM: The TSS and SM approaches are used to quickly recapture a lost target. Videos where the target suddenly moves or disappears briefly in the view range are limited in VOT2018, and thus the gain in EAO from TSS + SM is not obvious. However, the performance of the tracker with the LaSOT dataset is evident, and reaches 5%. This is attributed to the close to 10 times longer average video length in LaSOT compared to VOT2018, as well as more than 10 times higher number of frames containing the sudden movement or disappearance of the target from the field of view. More importantly, a speed of 50 FPS is achieved by our tracker via TSS + SM under global searching, which fully meets the real-time requirements.

**Figure 14:**
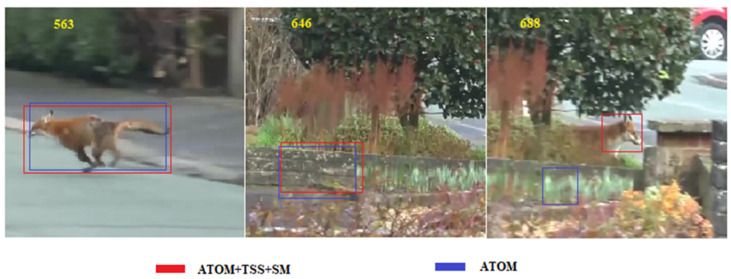
Effects of inclusion of TSS+SM on ATOM using video from VOT2018-LT.

TSS + SM is principally employed to enable the fast global search capability of our tracker. We used the ablation analysis results determined from the VOT2018 dataset. VOT2018 is a typical short-term tracking dataset, where the target rarely disappears from the field of view. Hence, the performance improvements from SM on this dataset are not significance. Our proposed TSS + SM can be easily integrated into other trackers. In the following, we describe an experiment based on the performance of ATOM for a video sequence from VOT2018-LT before and after the inclusion of TSS and SM (Figure 14). Prior to the addition of TSS and SM, ATOM is unable to capture rapidly the re-emerging target. However, following the inclusion of the proposed approach, ATOM demonstrates a fast global search capability that can quickly capture the re-emerging target.

### 4.4. Discussion

Employing EfficientNet-B0 as the backbone network results in a FLOPS of just 1/11, with the number of required parameters reduced to 1/5 of those used for the most popular backbone network, ResNet-50. This offers an innate advantage to our method. At present, numerous popular trackers employ Region Proposal Network (RPN) regression, yet they fail to perform well on hard samples such as those with largely varying target scales. The pre-defined anchor settings not only introduce ambiguous similarity scoring that severely hinder the robustness, but also require access to prior information relating to the data distribution [65]. We consider that the underlying concept of RPN hinders the generalization ability of the tracker. Thus, our target estimation component is trained to predict the DIoU between the target object and an estimated bounding box. We experimentally confirm the importance of global search capabilities in long-term tracking. We also include TSS + SM to allow for rapid global searching. In order to verify the effectiveness of our proposed framework in real scenarios, we provide Appendix A. At the same time, in order to verify that our method is data-driven, we provide Appendix B.

Since our method is the same as current mainstream methods, it also uses the appearance modelling method. Our method is prone to drifting when the target and extremely similar objects are occluded. Theoretically, there is an upper limit on discrimination for the trackers based on the appearance modelling method. In [65], a novel tracking framework that employs scene information is proposed, with dense local state vectors representing this information. The vector encodes the local area as a target, background or interference. These state vectors are propagated in the sequence and combined with the appearance model output to locate the target. Inspired by [65], our future research will also break through the limitations of pure appearance modelling.

## 5. Conclusions

In this paper, we design a tracker that is not only robust and accurate, but also has long-term and real-time capabilities. The EfficientNet-B0 significantly improves the feature extraction capacity of the backbone network and significantly reduces the network parameters, laying a solid foundation for rapid tracker speed. CBAM is applied to refine the features of the backbone network input before target classification, which can improve the discrimination of the tracker. In addition, we maximize the DIoU for accurate target estimation, increasing the convergence speed of the offline learning network and stabilizing the training process. We propose a novel two-stage search method combined with a screening module to achieve faster global searches. The tracker’s robustness is further improved via the interference filtering from the screening module. The successful performance of our method on five datasets validates the effectiveness of the proposed architectures. We also verify the effectiveness of each module through ablation analysis.

## Figures and Tables

**Table 1:** EfficientNet-B0 network structure.

Stage i	Operator F_i_	Resolution H_i_ × W_i_	Channels C_i_	Layers L_i_
1	Conv3 × 3	224 × 224	32	1
2	MBConv1, k3 × 3	112 × 112	16	1
3	MBConv6, k3 × 3	112 × 112	24	2
4	MBConv6, k5 × 5	56 × 56	40	2
5	MBConv6, k3 × 3	28 × 28	80	3
6	MBConv6, k5 × 5	14 × 14	112	3
7	MBConv6, k5 × 5	14 × 14	192	4
8	MBConv6, k3 × 3	7 × 7	320	1
9	Conv1 × 1&Pooling&FC	7 × 7	1280	1

**Table 2:** Comparison with the state-of-the-art tracking algorithms on theVOT2016 benchmark.

	SiamFC	SiamRPN	SiamDW	C-RPN	Ours
EAO	0.240	0.340	0.370	0.363	0.429
Robustness	0.460	0.260	0.240	-	0.186
Accuracy	0.530	0.560	0.580	0.594	0.609

**Table 3:** Comparison with the state-of-the-art tracking algorithms on theVOT2018 benchmark.

	STRCF	ECO	Ocean(Online)	SiamMask	ATOM	SiamRPN++	Ours
EAO	0.345	0.280	0.489	0.380	0.401	0.414	0.425
Robustness	0.215	0.276	0.117	0.276	0.204	0.234	0.198
Accuracy	0.523	0.484	0.592	0.609	0.590	0.600	0.606
Speed (fps)	2.9	3.7	25	35	30	35	52

**Table 4:** Comparison with the state-of-the-art tracking algorithms on theVOT2019 benchmark.

	Ocean (Online)	MAML	SiamBAN	SiamDW	Ours
EAO	0.350	0.295	0.327	0.242	0.346
Robustness	0.316	0.421	0.396	-	0.365
Accuracy	0.594	0.637	0.602	-	0.605

**Table 5:** Comparison with the state-of-the-art tracking algorithms on the GOT10K benchmark.

	Ocean (Online)	DiMP	SiamDW	Ours
AO	0.611	0.611	0.416	0.613
SR_0.5_	0.721	0.717	-	0.719

**Table 6:** Results of the ablation study for the proposed tracker using VOT2018.

BackBone	IoU/DIoU	CBAM	DAS	TSS+SM	VOT2018
ResNet-50	IoU				0.402
ResNet-50	IoU	√			0.406
ResNet-50	IoU		√		0.404
ResNet-50	IoU	√	√		0.408
ResNet-50	DIoU				0.404
ResNet-50	DIoU	√			0.407
ResNet-50	DIoU		√		0.405
ResNet-50	DIoU	√	√		0.412
ResNet-50	DIoU	√	√	√	0.413
EfficientNet-B0	IoU				0.410
EfficientNet-B0	IoU	√			0.416
EfficientNet-B0	IoU		√		0.414
EfficientNet-B0	IoU	√	√		0.419
EfficientNet-B0	DIoU				0.413
EfficientNet-B0	DIoU	√			0.417
EfficientNet-B0	DIoU		√		0.415
EfficientNet-B0	DIoU	√	√		0.424
EfficientNet-B0	DIoU	√	√	√	0.425

**Figure A1:**
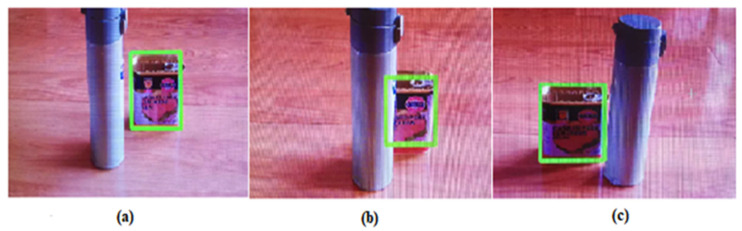
The performance of our tracker when using the camera to track in real time. (**a**) The situation when the target moves quickly to the left. (**b**) The situation when occlusion occurs. (**c**) The situation after occlusion.

## Appendix A

In order to more directly show the performance of our proposed tracker when using a camera to track a fast-moving target in real time, we provide Figure A1. From Figure A1, we can see that our tracker has good performance both when the target is moving fast and when occlusion occurs.

## Appendix B

The visual target tracking method based on deep learning is theoretically a data-driven method. The stronger the diversity of data used in training, the better the performance of our proposed method. We conducted ablation studies on the diversity of the data sources to determine the impact of data diversity on tracker performance. We chose four different training data settings, from low diversity to high diversity. Table A1 shows that the increase in diversity of the training data significantly improves the performance, which proves the ability of our method to utilize rich offline training data.

**Table A1:** The performance of the tracker on the VOT2018 dataset offline training using different scale data sets.

COCO	Youtube-BB	Tracking-Net	GOT10K	Accuracy	Robustness	EAO
√	×	×	×	0.520	0.223	0.396
√	√	×	×	0.588	0.215	0.405
√	√	√	×	0.600	0.203	0.421
√	√	√	√	0.606	0.198	0.425
